# Intelligent drugs based on notch protein remodeling: a defensive targeting strategy for tumor therapy

**DOI:** 10.1038/s41419-024-07008-7

**Published:** 2024-08-28

**Authors:** Yuliang Sun, Yilin Lu, Xinze Li, Yanan He, Then Kong Yong, Cheong Soon Keng, Badrul Yahaya, Yanli Liu, Juntang Lin

**Affiliations:** 1 Stem Cell and Biotherapy Technology Research Center, Xinxiang Medical University, Xinxiang, 453003 China; 2 Department of Biomedical Sciences, Advanced Medical and Dental Institute (AMDI), Universiti Sains Malaysia, SAINS@BERTAM, 13200 Kepala Batas, Penang Malaysia; 3 Breast Cancer Translational Research Program (BCTRP), Advanced Medical and Dental Institute, Universiti Sains Malaysia, Bertam, 13200 Kepala Batas, Penang Malaysia; 4 CryoCord Sdn Bhd, Bio-X Centre, 63000 Cyberjaya, Selangor Malaysia

**Keywords:** Targeted therapies, Drug delivery

## Abstract

In the process of tumor treatment, systemic drug administration is hindered by biological barriers, leading to the retention of a large number of drug molecules in healthy tissues and causing unavoidable side effects. The precise deployment of drugs at the tumor site is expected to alleviate this phenomenon. Here, we take endostatin and Her2 (+) tumors as examples and develop an intelligent drug with simple “wisdom” by endowing mesenchymal stem cells (MSCs) with an intelligent response program (iMSC^Endostatin^). It can autonomously perceive and distinguish tumor cells from non-tumor cells, establishing a logical connection between tumor signals and drug release. Enable it to selectively deploy drugs at the tumor site, thereby locking the toxicity of drugs at the tumor site. Unlike traditional aggressive targeting strategies that aim to increase drug concentration at the lesion, intelligent drugs are more inclined to be defensive strategies that prevent the presence of drugs in healthy tissues.

## Introduction

In the process of tumor treatment, therapeutic drugs administered intravascularly encounter multiple biological barrier systems, including intravascular barriers, endothelial barriers, and extracellular matrix barriers, before reaching the target site [1–3]. These barriers can hinder drug molecules from reaching the tumor site, exposing them to healthy tissues and causing unavoidable side effects [4]. For example, although endostatin has shown significant therapeutic effects in lung cancer, breast cancer, ovarian cancer, and lymphoma, it distributes easily to all tissues, with over 90%–95% of endostatin accumulating in normal tissues and organs [5–8]. And these endostatin, which accumulate in the heart, can cause cardiomyocyte cell apoptosis and mitochondrial dysfunction through Apaf-1 and p53 [9]. In clinical practice, approximately 6.38% of patients may experience varying degrees of adverse cardiac reactions, especially about 2.1% of patients who have to terminate treatment due to cardiac reactions [10].

Although traditional targeted therapy modalities such as antibody-drug conjugates and cell carriers can alleviate this phenomenon [11, 12], off-target effects are difficult to avoid, and a large number of drug molecules still struggle to penetrate these biological barriers. While antibody-drug conjugates can enhance the affinity of drug molecules to tumors, they often lack the ability to chemotactic towards the tumor site and the driving force to break through these biological barriers [13]. Cell carriers can recognize tumor-released cytokines through chemokine receptors on the cell membrane surface and deliver drugs to the tumor site [14–16]. However, the microenvironment of many diseases may have similar characteristics to the tumor microenvironment. For example, in viral myocarditis, similar to the tumor microenvironment, the chemokine CCL4 is overexpressed [17, 18]. In addition, the initial function of the receptors on the surface of the platform carried by cells is not to recognize the tumor microenvironment, and they may have difficulty effectively distinguishing the source of chemokines, which may lead to these transport platforms deviating from their targets or losing direction.

Traditional targeting emphasizes “aggressive strategies” to enrich more drug molecules in the lesion. However, we believe that, when drugs cannot entirely avoid off-target effects, strategies focusing on “defensive” to prevent the presence of drug molecules in healthy tissues outside of biological barriers may be more important. Therefore, it is crucial to achieve the intelligence of drugs so that they can autonomously distinguish/judge tumor cells and healthy tissue cells.

The native Notch signaling pathway is an important means of cell-to-cell communication [19, 20]. Previous studies have indicated that in the Notch protein, both the extracellular domain responsible for ligand recognition and the intracellular domain acting as a transcription factor can be replaced. Only the core region of the Notch protein needs to be retained, and it can still exert Notch-like functionality [20–22]. This modular signaling mechanism allows for the customization of input and output signals for cells, establishing logical connections between tumor recognition and response feedback signals (Release/deploy drugs).

In order to achieve the above goals, this study takes Her2 (+) tumors and endostatin as examples. By reconstructing the Notch protein for mesenchymal stem cells (MSCs), an artificial receptor for recognizing Her2 (+) tumor cells and a response structure for producing/deploying endostatin were customized to it, creating an intelligent drug based on MSCs (iMSC^Endostatin^). Experimental verification shows that iMSC^Endostatin^ exhibits extraordinary recognition/distinguishing ability for normal tissue cells from Her2 (+) tumor cells, which can precise deployment of endostatin at the tumor site, and locking treatment and drug toxicity to the lesion. Those iMSC^Endostatin^ that have not broken through the biological barrier will not produce and secrete endostatin due to their inability to receive tumor signals, thus avoiding the damage caused by off-target effects on healthy tissue cells in traditional targeting. In summary, The selective drug deployment of iMSC^Endostatin^ represents a defensive targeting strategy to reduce drug toxicity.

## Materials and methods

### Patients

This study was approved by the Ethics Committee of Xinxiang Central Hospital, and all patients were recruited from Xinxiang Central Hospital. Prior to participating in the study, all three patients provided written informed consent and agreed to the use of their samples for scientific research purposes. The study subjects were patients diagnosed with Her2-positive breast cancer, and all tumor tissue samples were evaluated using the immunohistochemistry (IHC) scoring system, with results of 3+.

### Cells and animals

The MSCs utilized in this study were used in this study were obtained from the Zhongyuan Stem Cell Research Institute of Xinxiang High Tech Zone. All cell lines were authenticated by short tandem repeat analysis, and mycoplasma tests were negative. All experimental procedures were conducted in accordance with the guidelines approved by the Ethics Committee of Xinxiang Medical University. Female Balb/c mice and Balb/c nude mice, aged 6–8 weeks (18–25 g), were procured from Vital River Laboratories (Beijing, China; license No. SCXK (Beijing) 2016-0006). Mice were randomly assigned to cages and housed in a specific pathogen-free (SPF) environment, with conditions set to a temperature of 25 °C, humidity of 50 ± 5%, and a 12-h light-dark cycle. In this study, the investigator was blinded to the group allocation of the animals during the experiment. No statistical method was used to predetermine the sample size for the mouse experiment, which was based on preliminary experimental results. No data were excluded from the analysis.

### Establishment of iMSC^Endostatin^ and fluorescence-labeled cells

The recognition structure of iMSC^Endostatin^ retains the core of Notch (Notch core), which is derived from the native Notch1 protein (nm_008714; Ile1427 to Arg1752). The extracellular and intracellular domains of the native Notch protein were replaced with Her2-affibody [23] and GAL4-VP64, respectively (recognition structure: Affibody-Notch(core)-GAL4-VP64). The sequence of the C-myc tag in the recognition structure is EQKLISEEDL. In the response structure (Gal4/UAS-Endostatin), we cloned five copies of the Gal4-DBD target sequence (GGAGCACTGTCCGAACG) into the minimal CMV promoter. The signal peptide (SA) sequence in the endostatin of the response structure is MALPVTALLPLALLLHAARP. The response structure contains a BFP tag. Therefore, the plasmid for the recognition structure is pLVX-Affibody-Notch(core)-GAL4-VP64, and the plasmid for the response structure is pHR-Gal4/UAS-Endostatin-IRES-mCherry-PGK-BFP. Subsequently, the recognition and response structures were integrated into the MSC genome via conventional lentiviral-mediated transfection.

BT474, Skov3, 293T, BJ, L02, Svog, HUVEC, and AC16 cells were labeled with GFP using lentivirus.

### Immunofluorescence and western blotting

Human breast cancer tissue: The slices were permeabilized with 0.5% Triton X-100 and blocked with 10% goat serum. The primary antibodies are rat anti-Her2 (MA1-82367; invitrogen), mouse anti-mmp-2 (AB3284; Abways), and rabbit anti-mmp-9 (MA5-32705; invitrogen). The secondary antibodies are goat anti-rat Cy5-labeled, mouse anti-mouse FITC-labeled and goat anti-rabbit Cy3-labeled IgG.

iMSC^Endostatin^: Permeabilization and blocking were conducted following the aforementioned procedure. The primary antibody is a mouse-derived c-myc tag (AB0001; Abways). The secondary antibody is Goat anti-mouse Fitc-labeled IgG.

WB: The protein samples were denatured in a metal bath at 95 °C and blocked with 5% nonfat milk. The primary antibodies are mouse anti-C-myc (ab56; Abcam), rabbit anti-Cyclin B1 (ET1608-27; HUABIO), rabbit anti-CDK2 (ET1602-6; HUABIO), rabbit anti-PCNA (ET1605-38; HUABIO), and mouse anti-GAPDH (60004-1; Proteintech). The secondary antibodies are goat anti-mouse IgG-HRP and goat anti-rabbit IgG-HRP.

### Recognition of Her2 (+) tumor by iMSC^Endostatin^

iMSC^Endostatin^ was co-cultured with BT474-GFP, Skov3-GFP, 293T-GFP, BJ-GFP, L02-GFP, Svog-GFP, HUVEC-GFP, and AC16-GFP cells in DMEM medium for 24 hours. Subsequently, confocal microscopy, live cell imaging, and flow cytometry analyses were conducted.

### Qualitative and quantitative analysis of endostatin production

Fluorescence analysis: iMSC^Endostatin^ and Skov3-GFP were inoculated into a 24-well plate coated with fibronectin for different co-culture times (15 min, 12 h, 24 h, 36 h, 48 h). Subsequently, cells were fixed with 4% paraformaldehyde (PFA), and cells exhibiting fluorescence changes were imaged under a microscope.

Microplate reader detection: iMSC^Endostatin^ and Skov3-GFP were co-cultured, and the mCherry signal was detected in the culture medium at 15 min, 12 h, 24 h, 36 h, and 48 h, respectively. Or iMSC^Endostatin^ was co-cultured with Skov3-GFP for 48 hours, and then the mCherry signal was detected every 4 hours.

ELISA: MSCs or iMSC^Endostatin^ will be co-cultured with Skov3-GFP for 48 h, and MSCs and iMSC^Endostatin^ cells will be sorted by flow cytometry and cultured in a 24-well plate coated with fibronectin for 24 h. The content of endostatin will be detected using an ELISA detection kit (CSB-E07973h; CUSABIO).

### Detection of vascular endothelial cell viability

Skov3h or AC16 cells were co-cultured with iMSC^Endostatin^ (without mCherry labeling) for 48 h using the medium composed of a 1:1 mixture of DMEM and ECM. The conditioned medium was then collected and concentrated 5-fold using an ultracentrifugation tube.

CCK-8: 8 × 10^3^ HUVEC cells were added into 96-well plates and cultured in different conditioned media for 48 h. The cell viability was detected using the CCK-8 assay.

CD105& WB: HUVEC cells were cultured in 6-well plates using different conditioned media for 48 h. Flow cytometry was conducted after incubation with CD105 antibody (51-9007648; BD Stemflow^TM^) for 30 min. Or the expression of C-myc, Cyclin B1, CDK2, and PCNA were detected by WB.

### Tube formation assay and matrigel plug assay

Tube formation assay: HUVEC cells were cultured with the aforementioned conditioned medium for 24 h (The experiment was divided into three groups: Control, Unactivated iMSC^Endostatin^, and Activated iMSC^Endostatin^). Subsequently, 50 µL of Matrigel was added to each well of a 96-well plate and HUVEC cells were incubated with the corresponding conditioned medium for 6 h. HUVEC cells were stained with calcein and imaged using a fluorescence microscope.

Matrigel plug assay: 200 µL Matrigel containing 50 µg bFGF was mixed with the above-conditioned medium and transplanted subcutaneously into female Balb/c nude mice (The experiment was divided into three groups: Control, Unactivated iMSC^Endostatin^, and Activated iMSC^Endostatin^, with each group consisting of 3 mice). After 9 days, the Matrigel was harvested for sectioning and CD34 staining (ab81289; abcam).

### Tumor recognition and antitumor effect of iMSC^Endostatin^ in vivo

Emt-6 cells were transfected with human Her2 protein and GFP by lentivirus.

Lung metastasis model: 2 × 10^6^ Emt-6-GFP cells were injected into female Balb/c mice through the tail vein. 3 × 10^6^ iMSC^Endostatin^ cells were injected into the tail vein 3 days after colonization (*n* = 5). After 48 h, the samples were harvested, sectioned, and imaged by a confocal microscope.

Solid tumor model: 1 × 10^6^ Emt-6-GFP cells were transplanted subcutaneously into mice, and 3 × 10^6^ iMSC^Endostatin^ cells were transplanted through the tail vein on day 11 (*n* = 5). After 48 h, tumors were harvested, sectioned, and imaged by confocal microscope.

Antitumor experiment: 1×10^6^ Emt-6 cells were subcutaneously transplanted into mice. The experiment was divided into three groups: The control group, the MSC group, and iMSC^Endostatin^ group (*n* = 5 mice per group). Subsequently, 3 × 10^6^ iMSC^Endostatin^ (without mCherry labeling)(iMSC^Endostatin^ group) or MSC (MSC group) cells were transplanted through the tail vein on the 4th, 7th, and 10th days, respectively. On the 14th day, the tumor was harvested and weighed, the volume was measured and CD34 immunofluorescence staining was performed.

### Cardiotoxicity

On the 14th day, ECG detection was performed on the aforementioned solid tumor mice. Serum was extracted for myocardial enzyme detection, and the heart was stained with hematoxylin and eosin (HE).

### Statistical analysis

Statistical analysis was performed with the SPSS 8.0 statistics software. All data were reported as mean ± standard deviation. All cell experiments were performed in triplicate at least. Dunnett’s test was used for comparisons among ≥3 groups. Statistical testing was conducted bilaterally, with a *p*-value < 0.05 to indicate statistical significance.

## Results

### Design principles of iMSC^Endostatin^

**Fig. 1:**
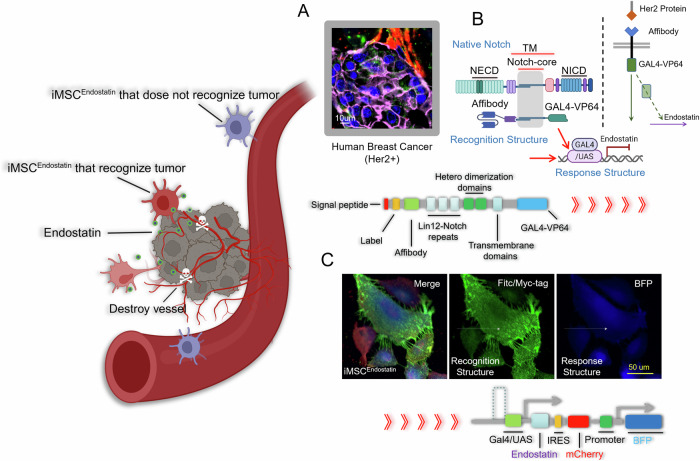
Design principles of iMSC^Endostatin^. **A** Human Her2 (+) breast cancer samples. Pink, green, and red fluorescence of the presence of Her2 protein, mmp-2, and mmp-9, respectively. **B** The structure of iMSC^Endostatin^ and its principle of responding to Her2 tumors. On the left side, display the structure of the native Notch protein and the structure of iMSC^Endostatin^. The structure of the native Notch protein includes the NECD (Notch extracellular domain), TM (transmembrane domain), and NICD (Notch intracellular domain). The recognition structure of iMSC^Endostatin^ includes Her2-affibody, TM, and GAL4-VP64. The response structure of iMSC^Endostatin^ mainly comprises the GAL4/UAS genetic regulatory elements and endostatin (therapeutic protein). On the right side is the process of iMSC^Endostatin^ responding to Her2 tumors: iMSC^Endostatin^ recognizes the Her2 protein on the tumor surface through its recognition structure, and subsequently, GAL4-VP64 transmits the tumor signal to initiate the expression of the drug-protein (endostatin). **C** Immunofluorescence images of the recognition structure and response structure of iMSC^Endostatin^.

Our study aimed to design an intelligent drug that can recognize/distinguish normal tissue cells from Her2(+) tumor cells, and selectively deploy the drug (in this study is endostatin) only at the tumor site. The Her2 protein is a prevalent clinical marker for malignant tumors and is often accompanied by the upregulation of matrix metalloproteinase (mmp)-2 and mmp-9, thereby promoting tumor proliferation and metastasis (Fig. 1A). The native Notch protein consists of three parts: Notch extracellular domain (NECD), Transmembrane domain (TM), and Notch intracellular domain (NICD) (Fig. 1B). Previous studies have confirmed that Notch proteins can still perform Notch-like functions even after their extracellular and intracellular domains are replaced. Her2 (+) tumors, we retained the core region of the Notch protein (nm_008714; Ile1427 to Arg1752), substituted its extracellular and intracellular domains with the Her2-affibody and GAL4-VP64, and introduced CD8α signal peptide (MALPVTALLLPLALLLHAARP) and C-myc tag (EQKLISEEDL) in turn at the N-terminus of Her2-affibody (Fig. 1B and Fig. S1). Subsequently, to enable iMSC^Endostatin^ to express endostatin, we designed a Gal4/UAS-endostatin structure in the response structure and added a BFP tag (Fig. 1B and Fig. S2). GAL4-VP64 in the iMSC^Endostatin^ can connect recognition and response structures and transform tumor signals into signals related to the expression and secretion of endostatin. Due to the preservation of the core region of the native Notch protein in this program, when iMSC^Endostatin^ recognizes Her2 (+) tumors, the γ-secretase in the cell can act on the S3 hydrolysis site of the core region, causing GAL4-VP64 to detach from the recognition structure and bind to the responsive structure, resulting in the expression of endostatin gene in iMSC^Endostatin^ (Fig. S3). The green fluorescence and blue fluorescence in Fig. 1C represent the recognition structure and response structure of iMSC^Endostatin^, respectively, which together constitute the intelligent response program of iMSC^Endostatin^.

### iMSC^Endostatin^ recognizes Her2 (+) tumors in vitro

**Fig. 2:**
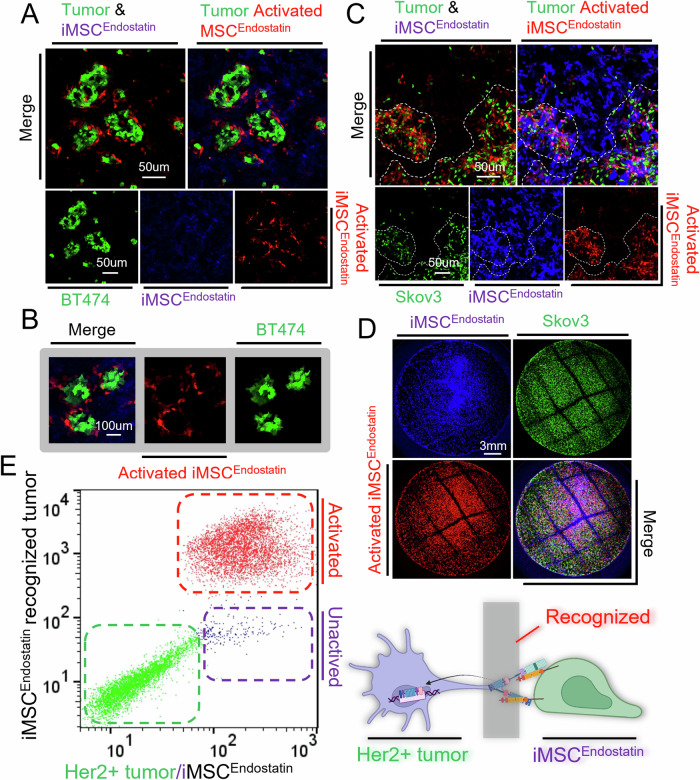
Intelligent Her2 (+) tumor recognition by iMSC^Endostatin^. **A**–**C** Accurate recognition of BT474 and Skov3 tumors by iMSC^Endostatin^. The green fluorescent cells represent BT474 or Skov3 cells. Red cells indicate iMSC^Endostatin^ that recognizes tumors, while blue cells indicate those that do not recognize tumors. **D** Macroscopic view of Her2 (+) tumor recognition by iMSC^Endostatin^. **E** Her2 (+) Tumor recognition by iMSC^Endostatin^ detected by flow cytometry.

The IRES structure guaranteed the co-expression of endostatin and mCherry in iMSC^Endostatin^. When iMSC^Endostatin^ recognizes the tumor, the cells will exhibit red fluorescence (mCherry). To assess iMSC^Endostatin^’s recognition capability for Her2 (+) tumors, two co-culture systems were established: BT474 (human breast cancer)—iMSC^Endostatin^ and Skov3 (human ovarian cancer)—iMSC^Endostatin^. The results revealed that iMSC^Endostatin^ showed extraordinary recognition of Her2 (+) tumor cells (Fig. 2A–C). In the BT474—iMSC^Endostatin^ co-culture system, iMSC^Endostatin^ (red), which recognizes BT474 (green), closely encompassed the tumor cell mass, while unactivated iMSC^Endostatin^ (blue) is observed where no recognition occurs (blue) (Fig. 2A, B). Similarly, in the Skov3-iMSC^Endostatin^ co-culture system, the iMSC^Endostatin^ that recognized Skov3 exhibited a distribution pattern similar to that of tumors (Fig. 2C). To further verify the reliability of the recognition of iMSC^Endostatin^, we divided the tumor area and non-tumor area in a macroscopic field of view with a diameter of 20 mm. As anticipated, iMSC^Endostatin^ accurately distinguishes between tumor and non-tumor sites and achieved exclusive deployment of endostatin (red) in the tumor area (Fig. 2D). Subsequent flow cytometry indirectly confirmed the superior recognition capacity of iMSC^Endostatin^ for Her2(+) tumors (Fig. 2E).

### Intelligent differentiation of tumor and normal tissue cells by iMSC^Endostatin^

**Fig. 3:**
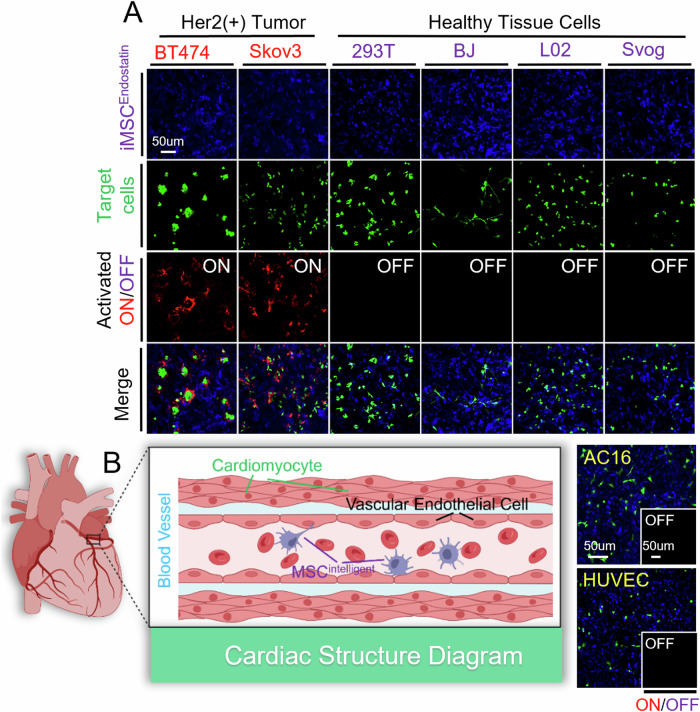
iMSC^Endostatin^ distinguishes between tumors and normal tissue cells. **A** iMSC^Endostatin^ was co-cultured with two types (BT474, Skov3) of tumors and four types of normal tissue cells, respectively (293T, BJ, L02, and Svog). **B** iMSC^Endostatin^ was co-cultured with cardiac-related cells, including vascular endothelial cells (HUVEC) and cardiomyocyte cells (AC16).

The intelligence of iMSC^Endostatin^ is reflected not only in its ability to recognize tumors but also in its ability to distinguish tumor tissue from normal tissue cells. To verify this property, two types of tumor cells (breast cancer and ovarian cancer) and four types of normal tissue cells (kidney, skin, liver, and ovary) were co-cultured with iMSC^Endostatin^. The results demonstrated that iMSC^Endostatin^ accurately distinguished between tumor and normal tissue cells, with activation occurring exclusively in Her2 (+) tumor cells and energy to the four types of normal tissue cells (Fig. 3A). Cardiotoxicity is the main side effect of endostatin, so it is necessary to prevent the deployment of endostatin in the heart. Considering the structure of the heart, cardiomyocyte cells (AC16) and vascular endothelial cells (HUVEC) were selected for co-culture with iMSC^Endostatin^. The results indicated that neither AC16 nor HUVEC could activate iMSC^Endostatin^ (Fig. 3B). The biological barrier hinders a large number of drugs from reaching the tumor site, leading to the drugs being distributed in the lesions and healthy tissues respectively. The recognition and distinguishing ability of iMSC^Endostatin^ to the tumor and normal tissue cells laid the foundation for its precise deployment of endostatin at the tumor site. This will avoid the toxic effects of drugs on healthy tissues and organs.

### iMSC^Endostatin^ deploys endostatin

**Fig. 4:**
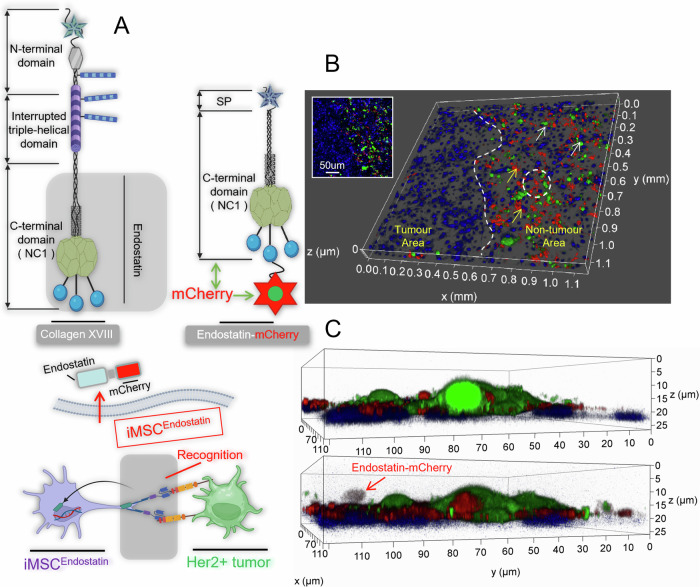
iMSC^Endostatin^ produces/deploys endostatin. **A** Schematic diagram of endostatin structure. We introduced the mCherry tag at the C-terminus of endostatin (iMSC^Endostatin^). **B** Confocal imaging of iMSC^Endostatin^ releasing endostatin. **C** 3D reconstructed image of **B** local. Arrows point to endostatin released by iMSC^Endostatin^.

**Fig. 5:**
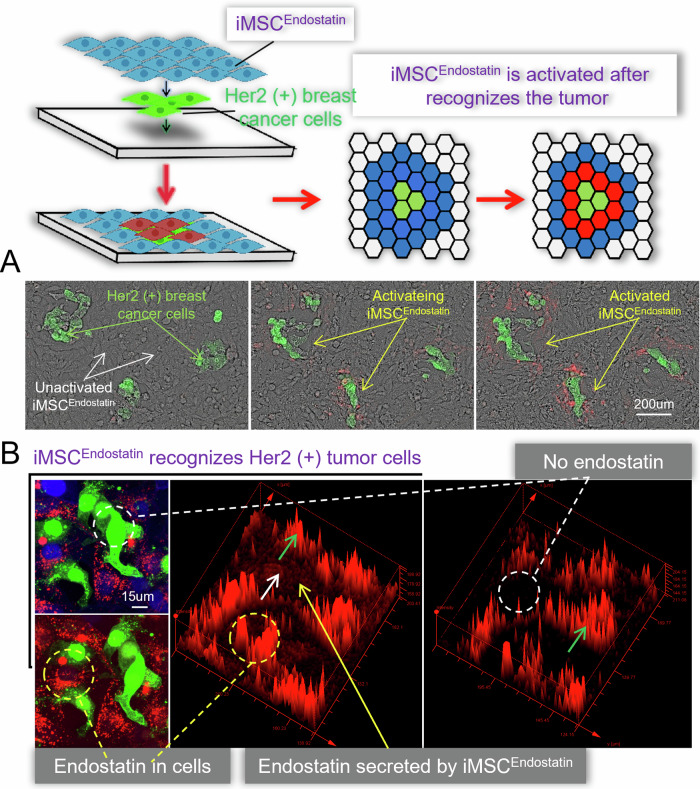
Dynamic display of iMSC^Endostatin^ deploying endostatin. **A** Live-cell workstation imaging of iMSC^Endostatin^ deploying endostatin. **B** 3D model fluorescence intensity imaging of iMSC^Endostatin^ release endostatin.

In this study, the drug released by iMSC^Endostatin^ upon recognition of tumors is endostatin. Since endostatin is a 20 kDa proteolytic fragment of the C-terminal noncollagenous domain (NC1) of type XVIII collagen, natural endostatin lacks a signal peptide structure. The deployment capability of iMSC^Endostatin^ refers to secreting its produced endostatin out of the cell. Therefore, we reconstructed the SP structure of endostatin in iMSC^Endostatin^. To verify the capacity of iMSC^Endostatin^ to produce and deploy endostatin, we removed mCherry from the response structure and fused it to the N-terminus of endostatin (iMSC^Endostatin^) (Fig. 4A). Under this structure, the red fluorescence signal will represent the endostatin produced by iMSC^Endostatin^, and subsequent confocal imaging confirmed the production capacity of iMSC^Endostatin^ (Fig. 4B). The phenomenon of endostatin-mCherry secretion and deployment by iMSC^Endostatin^ was further demonstrated through 3D imaging of Fig. 4B local (Fig. 4C). Subsequent live cell workstation imaging also dynamically showed iMSC^Endostatin^’s extraordinary recognition ability for Her2 (+) tumor cells and the phenomenon of deploying endostatin around the tumor (Fig. 5A and Video 1). For a more intuitive demonstration of iMSC^Endostatin^’s ability to secrete endostatin, we conducted 3D model fluorescence intensity imaging for Fig. 4C (Fig. 5B). The strong signal peak and weak signal peak in Fig. 5B correspond to the extracellular endostatin-mCherry that has not yet been secreted and the extracellular endostatin-mCherry that has been secreted, respectively. After washing with PBS, these weak signal peaks were lost in the endostatin-Skov3 co-culture system, which confirmed that these weak signal peaks originated from the secretion of iMSC^Endostatin^ into extracellular endostatin (Fig. 5B).

### Quantitative analysis of iMSC^Endostatin^’s deployment of endostatin

**Fig. 6:**
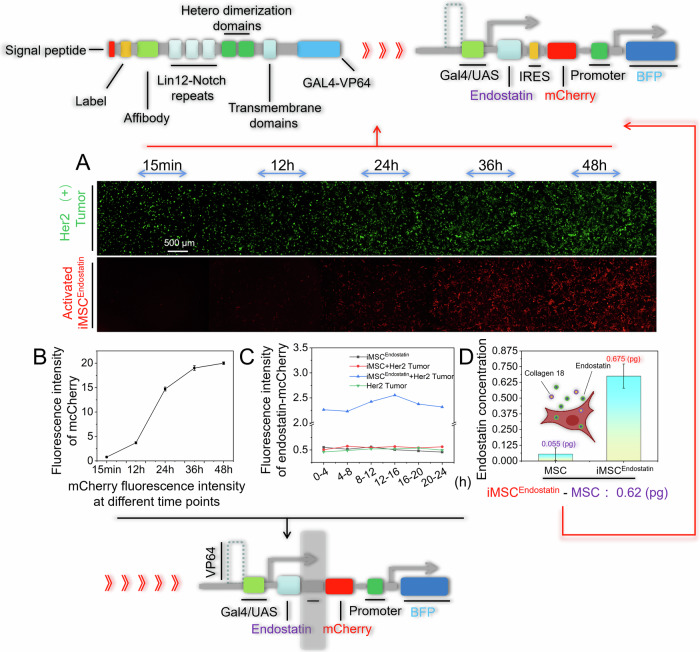
Quantitative analysis of iMSC^Endostatin^’s ability to deploy and produce endostatin. **A** Fluorescence intensity at various time points following iMSC^Endostatin^ recognition of tumors. **B** The endostatin-mCherry released by iMSC^Endostatin^ at different time points was detected by a microplate reader. **C** The microplate reader was utilized to detect the total amount of endostatin-mCherry accumulated every four hours following full activation of iMSC^Endostatin^. **D** The amount of released endostatin by iMSC^Endostatin^ was quantified using ELISA detection.

The quantitative analysis of the capacity of iMSC^Endostatin^ to produce and deploy endostatin indirectly evaluated its therapeutic potential. Since the IRES structure in iMSC^Endostatin^ enables the co-expression of endostatin and the mCherry gene, the accumulated red fluorescence intensity in iMSC^Endostatin^ will reflect the expression of endostatin. Through fluorescence imaging of iMSC^Endostatin^-Her2 (+) tumor cells co-culture system, we can conclude that iMSC^Endostatin^ can start to produce endostatin within 12 h after recognizing Her2 (+) tumor cells and reach the peak at 48 h (Fig. 6A). This conclusion was further supported by the fluorescence intensity of endostatin-mCherry accumulated every 12 h in the medium of iMSC^Endostatin^-Her2 (+) tumor cells co-culture system (Fig. 6B). Moreover, once the production of endostatin reaches a peak, iMSC^Endostatin^ can continuously and stably produce endostatin, and similar amounts of endostatin-mCherry will be secreted into the medium every four hours (Fig. 6C). Given the similar domains of natural collagen XVIII and endostatin, to determine the concentration of endostatin, we subtracted the ELISA signal of the naïve MSCs group from that of the iMSC^Endostatin^ group to exclude the interference of collagen XIII. The results indicated that every 5×10^4^ activated iMSC^Endostatin^ cells produced approximately 0.62 pg of endostatin every 24 h (Fig. 6D). Notably, in clinical practice, the number of stem cells injected intravenously can reach hundreds of millions, and the cells can survive in animal models for more than 6 months [24, 25]. The release of drug molecules is a specific response of iMSC^Endostatin^ to received tumor signals, which will lock the toxicity of drugs at the tumor site. Although the process of iMSC^Endostatin^ processing tumor signals is a slow response, it can continuously and selectively produce endostatin in high yields at the tumor site after being fully activated.

### iMSC^Endostatin^ inhibits angiogenesis in vitro

**Fig. 7:**
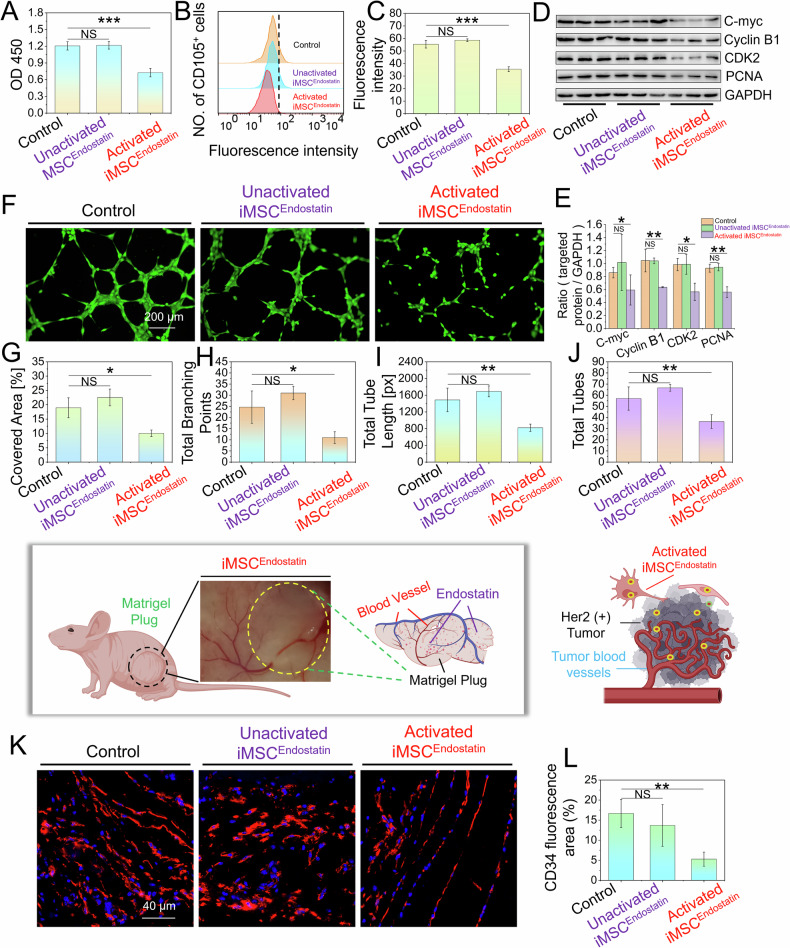
iMSC^Endostatin^ inhibits angiogenesis in vitro. **A** HUVEC cell viability was assessed using the CCK-8 assay. **B**, **C** The effect of activated iMSC^Endostatin^ on the expression of CD105 in HUVEC was detected by flow cytometry. **D**, **E** Proliferation-related protein expression levels, including C-myc, Cyclin B1, CDK2, and PCNA in HUVEC, were assessed using WB. The relative expression of targeted proteins was quantified using ImageJ. The original blots corresponding to these experiments are displayed in Fig. S4. **F**–**L** The ability of activated iMSC^Endostatin^ to inhibit vascular formation in vitro and in vivo was evaluated through tube formation assay and matrigel plug assay, respectively.

The antitumor activity (in this article is inhibition of angiogenesis) of intelligent drugs originates from the drug proteins (in this article is endostatin) expressed by response structure. In vitro, the co-culture system of iMSC^Endostatin^-Her2 (+) tumor cells and iMSC^Endostatin^-AC16 cells (cardiomyocytes) can simulate iMSC^Endostatin^ (recognition) activated at the tumor site and iMSC^Endostatin^ (non-recognition) unactivated in healthy tissue, respectively. In the CCK-8 assay, we observed that only the activated iMSC^Endostatin^, which recognizes tumors, can inhibit the activity of vascular endothelial cells. In contrast, iMSC^Endostatin^ that is not activated by cardiomyocytes (non-tumor cells) does not significantly affect the activity of vascular endothelial cells (Fig. 7A). CD105 is highly expressed in reshaped or newly formed blood vessels and can accurately reflect the proliferation status of endothelial cells and serve as a typical marker for evaluating the proliferation of vascular endothelial cells. Flow cytometry revealed that only activated iMSC^Endostatin^ significantly inhibited the expression of CD105 in HUVEC (Fig. 7B, C), and consistent results were obtained via WB (Fig. 7D, E). Additionally, the tube formation assay is an important experiment for simulating vascular formation in vitro. Our results further demonstrated that the covered area, total branching points, total tube length, and total tubes in the activated iMSC^Endostatin^ group were significantly lower than those in the control group (Fig. 7F–J), which strongly suggested that activated iMSC^Endostatin^ significantly inhibits angiogenesis in vitro. Consistently, in the subsequent matrigel plug assay, the fluorescence area of CD34+ cells in the activated iMSC^Endostatin^ group was also significantly lower than that in the control group (Fig. 7K, L). In conclusion, cell sensing/response pathways customized for iMSC^Endostatin^ can enable them to autonomously sense user-specified disease or injury signals, and selectively deploy therapeutic functions at tumor sites.

### Tumor recognition and antitumor effect of iMSC^Endostatin^ in vivo

**Fig. 8:**
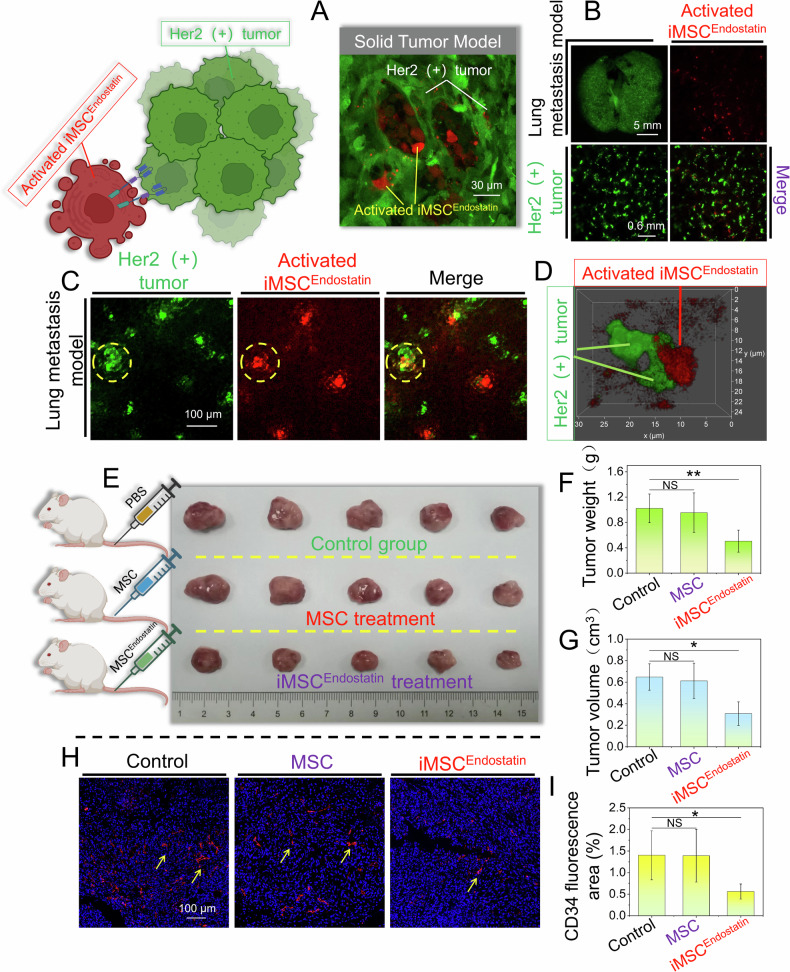
The In vivo recognition of Her2 (+) tumors and the antitumor effects of iMSC^Endostatin^. **A** Recognition of solid tumors by iMSC^Endostatin^. **B**–**D** Recognition of tumors by iMSC^Endostatin^ in lung metastasis models. **E**–**G** Images of tumor in model mice after iMSC^Endostatin^ treatment, as well as tumor weight and volume. **H**, **I** CD34 staining of tumor sections in model mice after iMSC^Endostatin^ treatment.

**Fig. 9:**
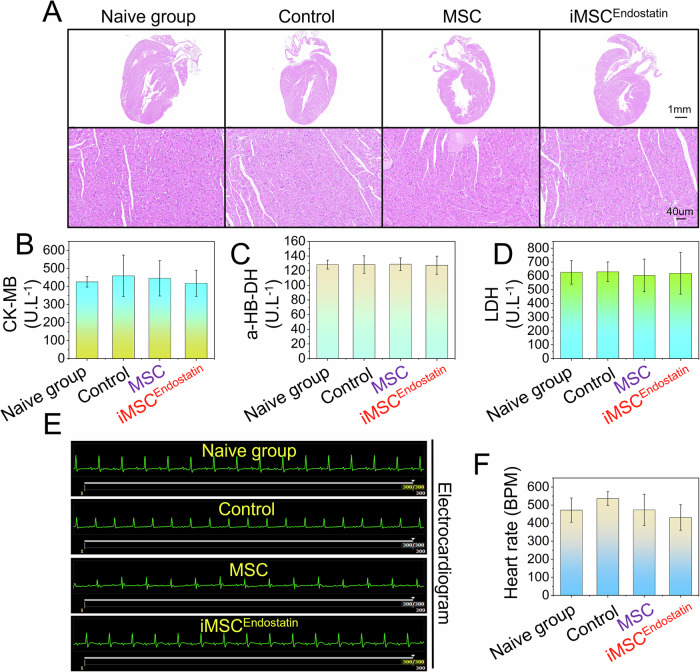
Detection of cardiotoxicity induced by iMSC^Endostatin^. **A** Morphological changes in the heart of model mice after iMSC^Endostatin^ treatment by hematoxylin and eosin staining. **B**–**D** Detection of myocardial enzymes in model mice. **E**, **F** ECG monitoring of model mice.

Intelligent drugs are based on MSCs as the main structure and previous studies have demonstrated that the chemotaxis of MSCs facilitates them to perceive chemotactic signals and penetrate into tumor tissue [26]. To verify the ability of iMSC^Endostatin^ to recognize Her2(+) tumor cells and its efficacy in inhibiting tumor growth in vivo, we established solid Her2(+) tumor and lung metastasis models. The results indicate that even within the in vivo context, the intelligent response program of iMSC^Endostatin^ remains operational and allows the recognition of Her2(+) tumors and the administration of endostatin (Fig. 8A–C). Moreover, only a small number of cells were required to fully activate iMSC^Endostatin^ (Fig. 8D). This may also enable iMSC^Endostatin^ to play an advantage in micrometastasis lesions. After the transplantation of iMSC^Endostatin^ (without mCherry labeling), significant inhibitory effects were observed on the weight (Fig. 8F), volume (Fig. 8E, G), and vascular density of the tumor (Fig. 8H, I) in model mice. Nevertheless, since natural MSCs do not contain an intelligent response program that drives endostatin, they cannot inhibit the formation of blood vessels and the progression of tumors (Fig. 8E–I). More importantly, consistent with our design expectations, there were no significant cardiotoxic reactions observed during iMSC^Endostatin^ treatment. The heart structure (Fig. 9A), serum myocardial enzyme concentration (Fig. 9B–D), electrocardiogram (Fig. 9E), and heart rate (Fig. 9F) in the treatment group did not significantly differ from those in the control group.

## Discussion

Drug molecules distributed in healthy tissues due to the hindrance of biological barriers can cause potential toxic side effects [4]. Although traditional targeting strategies can increase the enrichment ability of drugs at the tumor site, they still cannot avoid a large number of drugs remaining in normal tissues [27–30]. Therefore, there is an urgent need for a drug-targeting approach capable of avoiding the presence of drug molecules in healthy tissues while completing treatment. Here, we introduce a completely different drug form using Endostatin and Her2 (+) tumors as an example: intelligent drug (iMSC^Endostatin^). In this study, we reconstructed the Notch protein in MSC cells, enabling iMSC^Endostatin^ to acquire the ability to perceive/distinguish tumor and non-tumor cells and selectively deploy drugs (Figs. 2 and 4).

Intelligent drugs exhibit several distinct features over traditional drugs. Firstly, iMSC^Endostatin^ is an intelligent drug with simple “wisdom”. Its intelligence is reflected in the recognition and distinguishing of tumor cells from healthy tissue cells. Through reshaping cellular behavior, intelligent drugs can convert recognized tumor signals into the production/secretion of drugs, establishing a logical connection between the two. This allows iMSC^Endostatin^ to deploy drugs only at tumor sites, thereby confining/locking treatment and drug toxicity to the tumor site. Therefore, even though biological barriers can isolate drugs outside the lesion, unactivated iMSC^Endostatin^ in healthy tissues does not deploy any drug molecules. Pursuing defense is the core advantage of intelligent drugs, which differs from traditional targeting strategies that focus on increasing drug concentration within lesions; intelligent drugs focus more on removing drugs outside lesions. Secondly, the intelligent drugs (iMSC^Endostatin^) is composed of MSCs and an intelligent response program (Fig. 1C). The advantage of MSCs is that they can have the ability to home to tumors like T cells, but unlike T cells, they can avoid tumor-induced immunosuppressive blockade [26, 31]. Moreover, the response program in iMSC^Endostatin^ operates independently of the cell’s original structural proteins, freeing it from constraints imposed by the MSCs’s endogenous system. The expression/secretion of endostatin in iMSC^Endostatin^ is solely regulated by the tumor signals received by iMSC^Endostatin^ (Fig. 3B). Lastly, iMSC^Endostatin^ serves as both a drug and a “drug factory”. Therefore, Once the recognition of the tumor is completed, it can continuously produce and secrete endostatin in high yield (Fig. 6C, D), which can greatly shorten the treatment cycle and frequency.

**Fig. 10:**
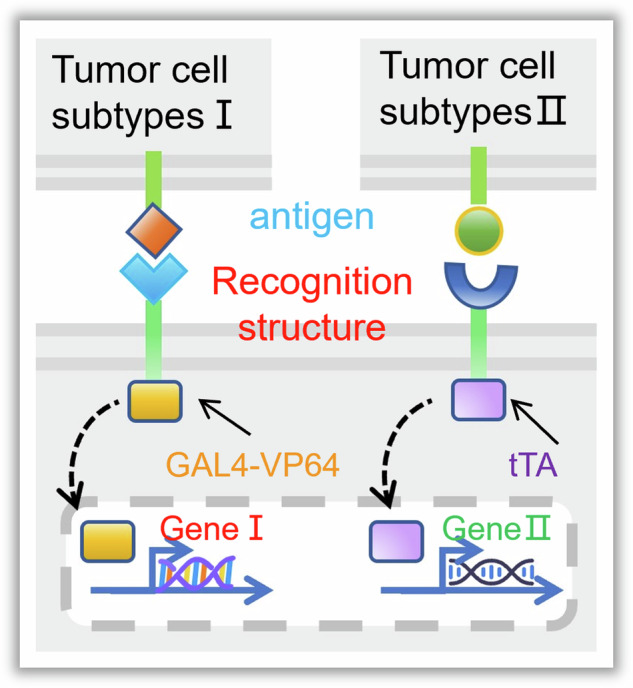
Design scheme for introducing multiple response programs in iMSC^Endostatin^.

Due to the plasticity of its DNA-driven response program and the modular structure of the Notch protein [21, 32], intelligent drugs can predefine different input signals and output signals (Figs. 2 and 3). For example, by replacing the antigen receptor in the recognition structure to change the signal type recognized by intelligent drugs (target tumor type), or by changing the drug sequence in the response structure to enable iMSC^Endostatin^ to output different drug types (Fig. 10). This enables users to design different drugs for personalized treatment according to different types of tumors.

Furthermore, the Notch pathway possesses a direct and simple signal transduction mechanism, and the signal transduction in its core region is triggered by the physical tension generated between cells [21, 33]. Therefore, the recognition of tumors by intelligent drugs only depends on their contact with it and is not regulated by various cytokines. This mechanism prevents the tumor from remotely activating intelligent drugs, thereby averting endostatin leakage. In addition, applying different signal transduction structures (such as Gal4-VP64 in the article) is expected to simultaneously introduce multiple sets of different response programs into intelligent drugs, giving them more “intelligence” to perform more complex actions (Fig. 10). In conclusion, the precise deployment of iMSC^Endostatin^ for drug toxicity is an effective means to improve the side effects of drugs caused by biological barriers. This defensive targeting strategy provides a new option for cancer treatment.

### Supplementary information

Supplemental Legends and Figures

Original WB results

The recognition of Her2 tumors by iMSC^Endostatin^ and its precise deployment of endostatin around the tumor.

## Data Availability

The raw data generated in this study are available upon request from the corresponding authors.
