# Supplementary material for: Intelligent drugs based on notch protein remodeling: a defensive targeting strategy for tumor therapy
Source: Cell Death Dis. 2024 Aug 28;15(8):632. doi: 10.1038/s41419-024-07008-7 (PMC11358381; doi:10.1038/s41419-024-07008-7)
Supplement: Supplementary file 1 — Supplemental Legends and Figures [file 41419_2024_7008_MOESM1_ESM.docx]

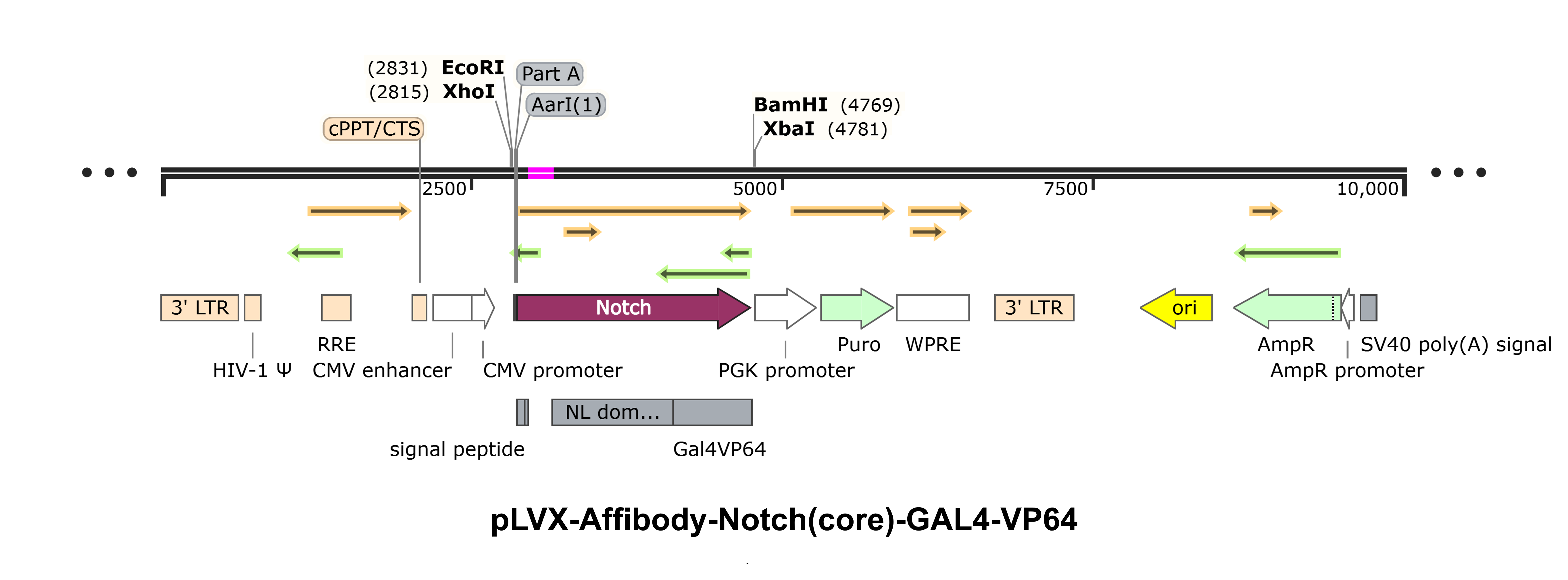


**Fig. S1 Plasmid map of recognition structure of iMSC^Endostatin^**


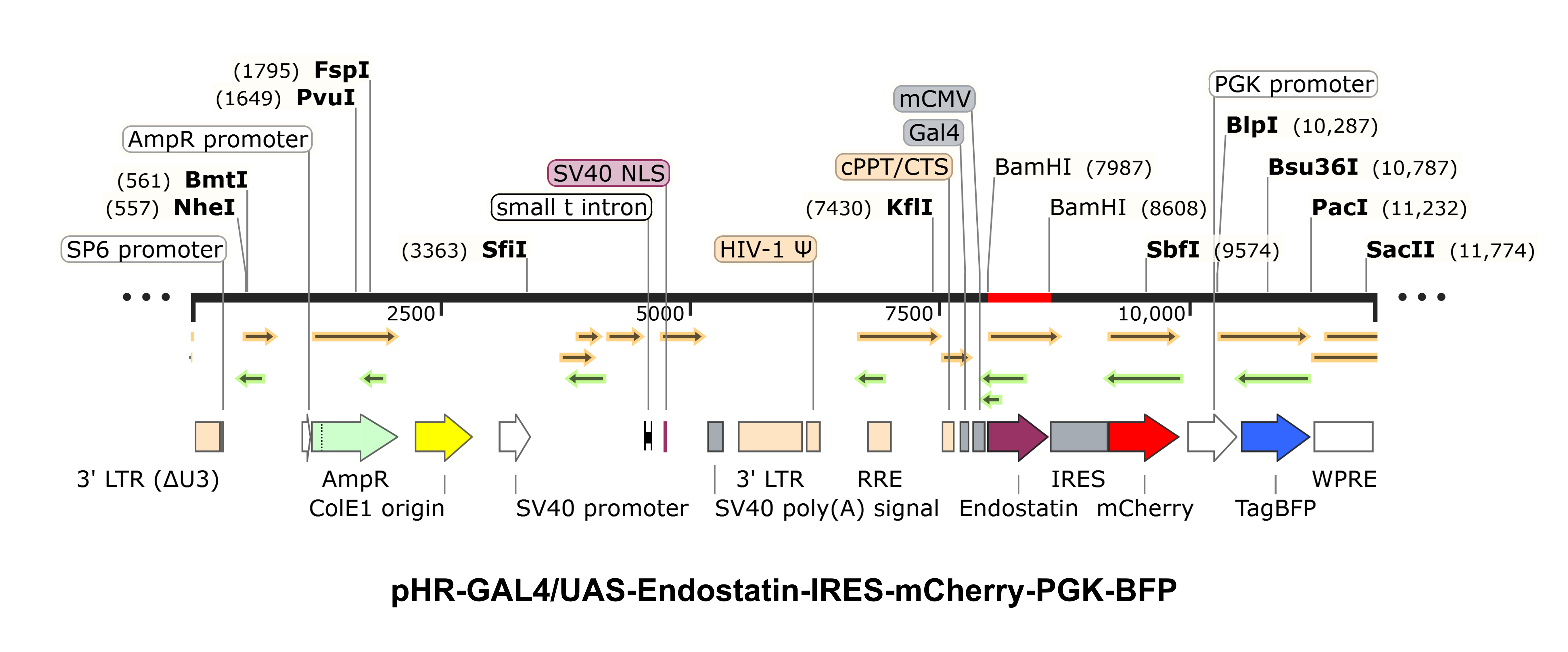


**Fig. S2 Plasmid map of response structure of iMSC^Endostatin^**


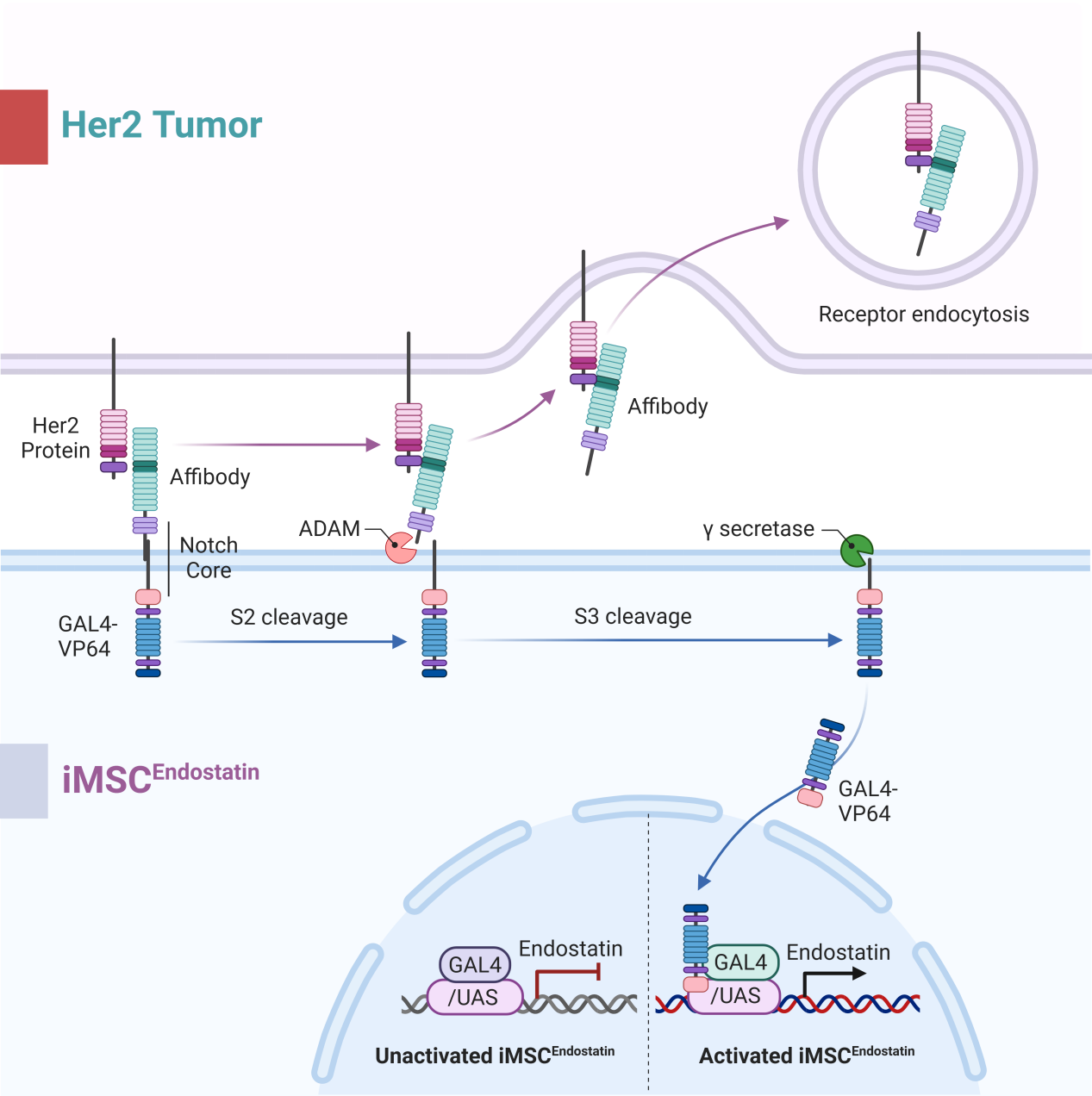


**Fig. S3 Illustration of the intelligent activation mechanism of iMSC^Endostatin^ recognizing Her2 tumors.** When iMSC^Endostatin^ recognizes the Her2 protein of the tumor, its recognition structure (Affibody-Notch (core)-GAL4-VP64) first undergoes cleavage at the S2 site (within the Notch core) by the ADAM enzyme. The resulting fragment is then cleaved at the S3 site (within the Notch core) by γ-secretase, releasing the intracellular segment GAL4-VP64. Subsequently, GAL4-VP64 translocates to the nucleus and binds to the responsive element, leading to the expression of endostatin (drug protein).
