# Supplementary figures and images for: Intelligent drugs based on notch protein remodeling: a defensive targeting strategy for tumor therapy
Source: Cell Death Dis. 2024 Aug 28;15(8):632. doi: 10.1038/s41419-024-07008-7 (PMC11358381; doi:10.1038/s41419-024-07008-7)

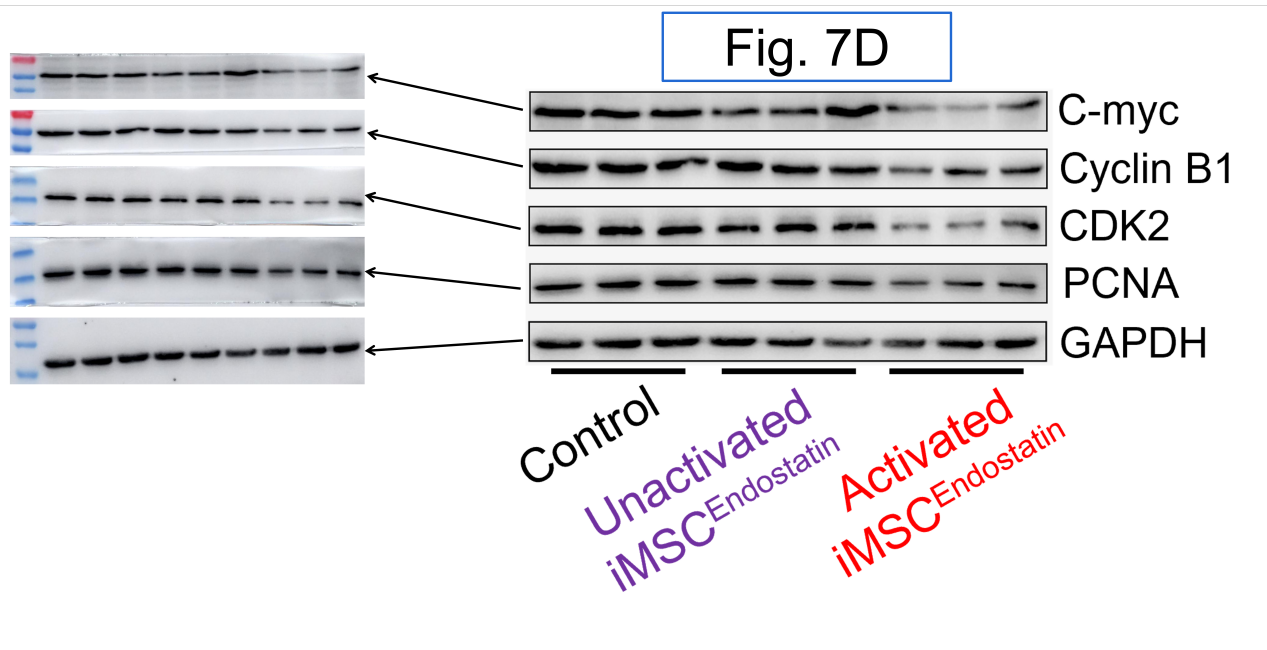


**Fig. S4 The original western blot images for Fig. 7D.**

Supplement: Supplementary file 2 — Original WB results [file 41419_2024_7008_MOESM2_ESM.docx]
